# Survival nomogram for endometrial cancer with lung metastasis: A SEER database analysis

**DOI:** 10.3389/fonc.2022.978140

**Published:** 2022-10-07

**Authors:** Guangwei Yan, Yingbin Li, Yanmin Du, Xiaotian Ma, Yifei Xie, Xianxu Zeng

**Affiliations:** Department of Pathology, The Third Affiliated Hospital of Zhengzhou University, Zhengzhou, China

**Keywords:** endometrial cancer, lung metastasis, overall survival, nomogram, SEER

## Abstract

**Purpose:**

The lung is the most common distant metastatic organ in patients with endometrial cancer (EC) but is rarely reported. This study examines the association between clinical characteristics and overall survival (OS) in EC with lung metastasis.

**Methods:**

Patients with EC who had accompanying lung metastasis were selected from the Surveillance, Epidemiology, and End Results (SEER) database between 2010 and 2017. Univariate and multivariate Cox regression were used to estimate hazard ratios (HRs) and 95% confidence intervals (95% CIs) and assess OS outcomes related to EC with lung metastasis. A Cox proportional hazards nomogram model for OS was constructed and validated. The calibration plot, receiver operating characteristic (ROC) curve and decision curve analysis (DCA) were used to evaluate the discriminative ability and clinical benefit of the novel nomogram. Kaplan–Meier curves and scatter diagram analysis were used to investigate the risk stratifications of the nomogram.

**Results:**

Overall, 1542 EC patients with lung metastasis between 2010 and 2017 were included and randomly divided into training and validation cohorts. A nomogram model was constructed using the clinical characteristics of tumor grade, histological type, surgery, adjuvant chemotherapy, adjuvant radiation, brain metastasis and liver metastasis. The concordance indexes (C-indexes) were 0.750 (95% CI, 0.732-0.767) and 0.743 (95% CI, 0.719-0.767) for the training cohort and validation cohort, respectively. Calibration plots and DCA showed good clinical applicability of the nomogram. The areas under the curves (AUCs) were 0.803 and 0.766 for 1-year and 3-year OS, respectively, indicating that the nomogram model had a stable discriminative ability. An online calculator of our nomogram is available on the internet at https://endometrialcancer.shinyapps.io/DynNomapp/. Additionally, patients in the high-risk group had a significantly worse OS than those in the low-risk group.

**Conclusion:**

An easy-to-use, highly accurate nomogram was developed for predicting the prognosis of EC patients with lung metastasis.

## Introduction

Endometrial cancer (EC) is the most common gynecologic malignancy in developed countries, with approximately 66,570 new cases in the United States in 2021, and the incidence continues to increase by approximately 1% per year (1). According to its frequent symptoms at an early stage, EC is often diagnosed at FIGO stages I or II (75%). Although most cases are often detected early, approximately 10% to 15% of women presenting with advanced or metastatic disease have a poor prognosis (2).

Stage IVB EC patients presenting with distant metastatic disease (including inguinal lymph node, intraperitoneal disease, lung, bone, brain, or liver) have a poor prognosis. The incidence of stage IVB disease is approximately 5–10%, with a 5-year overall survival (OS) of less than 10%, despite the quality of multimodality therapy available (3, 4).

Survival is dependent on other predictive factors. Abu-Rustum devised a nomogram based on five simple criteria (age, negative lymph nodes, FIGO stage, histological subtype and grade) to predict the OS of EC patients with a high concordance probability (5). Lymph node metastasis remains an important prognostic factor of EC; however, distant metastasis involving the liver, brain, bone or lung is rare. The lung is the most common extraperitoneal organ metastasis in EC, with an incidence of 1.5% (6, 7). Several retrospective studies have focused on management strategies and prognostic significance (8–10), but no predictive model for the risk and prognostic factors for EC with lung metastasis has been established. The objective of this population-based cohort study was to analyze the clinical features and assess the risk factors for EC with lung metastasis and to establish an independent nomogram for predicting the poor survival of EC with lung metastasis by using the Surveillance, Epidemiology, and End Results (SEER) database.

## Materials and methods

### Patient selection and data source

The data of women diagnosed with EC were obtained from the statewide SEER Program of the National Cancer Institute and queried using SEER*Stat software (version 8.3.5). The SEER database is the largest population‐based tumor registry in the United States, and nearly 97% of all incident cancer cases are captured within the registry area (11). Patients were identified using the International Classification of Diseases (ICD) code O-3 system (endometrium: C54.1) for primary tumor sites between 2010 and 2017. The histological types included for analysis were endometrial endometrioid adenocarcinoma (EEA), serous endometrioid adenocarcinoma (SEA), and other types in accordance with ICD-O-3 morphological codes (12) (**Table S1**). We included only patients after 2010 as defined by the SEER Combined Mets at DX-lung because information concerning lung metastasis was available from 2010.

In this study, clinical and pathological variables included age at diagnosis, race, histological type, tumor size, tumor grade, marital status, lymphadenectomy and distant metastasis (lung, liver, bone and brain). Adjuvant treatment included radiotherapy, chemotherapy, and surgery of the primary site recorded by program coding as defined by SEER. Tumor grades were defined as I (well differentiated), II (moderately differentiated), and III/IV (poorly differentiated or undifferentiated). For survival analyses, we collected information regarding the survival months, cause of death and cancer-specific mortality.

### Statistical analysis

All eligible patients were randomly divided into training and validation cohorts at a ratio of 7:3. The distribution of demographic and clinical variables was compared in the two cohorts using chi-square tests, and medians with interquartile ranges (IQRs) are used to describe continuous variables.

In the follow-up statistics, we observed that among the 1542 enrolled patients, the numbers of death cases attributable to any cause and to EC were 96 and 1274, respectively; therefore, the primary outcome measure was OS in our study. OS was defined as the time interval between EC with lung metastasis diagnosis and death from any cause (all‐cause). Univariate Cox regression was used to estimate the hazard ratios (HRs) and 95% confidence intervals (95% CIs) to determine the statistically significant risk factors related to OS. Variables with a P value <0.05 in univariate analysis were incorporated into multivariate Cox regression analysis using a stepwise selection process with the Akaike information criterion (AIC).

Next, the model with prognostic factors was used to construct a prognostic nomogram. The calibration curves based on the bootstrap method and decision curve analysis (DCA) curves at 12 and 36 months were used to validate the predictive accuracy and clinical applicability of the nomogram. The discrimination of the nomogram was quantified using time-dependent receiver operating characteristic (ROC) curve analysis for 12 and 36 months in the training and validation cohorts. The area under the curve (AUC) is a widespread metric used to evaluate classification models, especially in medical science. In addition, according to the median risk score by using the nomogram, all patients were divided into high- and low-risk groups. Kaplan–Meier and scatter diagram analyses were used to plot survival curves for group comparisons and to assess the significance of differences in event rates with the log-rank test in the training and validation cohorts (13).

All statistical analyses were conducted with R software (version 3.6.1). Two-sided hypothesis tests were used for statistical analysis, and *P* < 0.05 was considered statistically significant.

## Results

### Study population

Overall, 1542 EC patients with lung metastasis between 2010 and 2017 from the SEER database were included. Patient demographic and clinical characteristics are summarized in **Table 1**. The median age was 65 years (IQR 58–72). The majority of patients were white (72.0%), with diagnoses of EEA (54.5%) and grade III/IV disease (51.4%). Of the 1542 patients, 296 (19.2%) presented with liver metastases, 261 (16.9%) with bone metastases, 81 (5.3%) with brain metastases, and 152 (9.86%) with more than three metastatic organs. Surgery was performed in 698 (45.3%) women. With respect to adjuvant therapy, 956 (62%) patients were treated with chemotherapy, and 395 (25.6%) were treated with radiotherapy. In addition, there were no statistically significant differences in the demographic or clinical characteristics between the training and validation cohorts (all *P* > 0.05). A total of 1274 patients died after the follow-up cutoff date, and only 96 patients (7.53%) died due to causes other than EC. Notably, the prognosis of EC patients with lung metastasis was very poor, and the median survival time in these patients was only 8 months (IQR 2–19), with 1-year and 3-year OS rates of 38.06% and 9.86%, respectively.

**Table 1:** Demographic and clinical characteristics of EC patients with lung metastasis.

	Training cohort	Validation cohort	Overall	χ2	*P*
	(N=1079)	(N=463)	(N=1542)		
Age(year)				0.197	0.657
≤65	563 (52.2%)	248 (53.6%)	811 (52.6%)		
>65	516 (47.8%)	215 (46.4%)	731 (47.4%)		
Race				1.759	0.415
White	766 (71.0%)	344 (74.3%)	1110 (72.0%)		
Black	206 (19.1%)	78 (16.8%)	284 (18.4%)		
Other	107 (9.9%)	41 (8.9%)	148 (9.6%)		
Marital status				4.944	0.084
Married	452 (41.9%)	222 (47.9%)	674 (43.7%)		
Single	247 (22.9%)	98 (21.2%)	345 (22.4%)		
Other	380 (35.2%)	143 (30.9%)	523 (33.9%)		
Grade				4.776	0.311
I	62 (5.7%)	26 (5.6%)	88 (5.7%)		
II	98 (9.1%)	39 (8.4%)	137 (8.9%)		
III/IV	537 (49.8%)	255 (55.1%)	792 (51.4%)		
Unknown	382 (35.4%)	143 (30.9%)	525 (34.0%)		
Histological type				4.741	0.192
EEA	601 (55.7%)	240 (51.8%)	841 (54.5%)		
SEA	164 (15.2%)	70 (15.1%)	467 (30.3%)		
Others	314 (29.1%)	153 (33.0%)	286 (18.5%)		
Tumor size (cm)				3.295	0.348
<5	161 (14.9%)	64 (13.8%)	225 (14.6%)		
5-10	252 (23.4%)	121 (26.1%)	373 (24.2%)		
>10	125 (11.6%)	63 (13.6%)	188 (12.2%)		
Unknown	541 (50.1%)	215 (46.4%)	756 (49.0%)		
Surgery				0.008	0.892
No	590 (54.7%)	254 (54.9%)	844 (54.7%)		
Yes	489 (45.3%)	209 (45.1%)	698 (45.3%)		
Radiotherapy				0.058	0.809
No	805 (74.6%)	342 (73.9%)	1147 (74.4%)		
Yes	274 (25.4%)	121 (26.1%)	395 (25.6%)		
Chemotherapy				1.458	0.227
No	399 (37.0%)	187 (40.4%)	586 (38.0%)		
Yes	680 (63.0%)	276 (59.6%)	956 (62.0%)		
Brain metastasis				0.086	0.769
No	1024 (94.9%)	437 (94.4%)	1461 (94.7%)		
Yes	55 (5.1%)	26 (5.6%)	81 (5.3%)		
Liver metastasis				0.112	0.737
No	869 (80.5%)	377 (81.4%)	1246 (80.8%)		
Yes	210 (19.5%)	86 (18.6%)	296 (19.2%)		
Bone metastasis				0.578	0.447
No	902 (83.6%)	379 (81.9%)	1281 (83.1%)		
Yes	177 (16.4%)	84 (18.1%)	261 (16.9%)		
Lymph nodes resected				0.035	0.852
No	842 (78.0%)	364 (78.6%)	1206 (78.2%)		
Yes	237 (22.0%)	99 (21.4%)	336 (21.8%)		

EEA, endometrial endometrioid adenocarcinoma; SEA, serous endometrioid adenocarcinoma.

### Independent prognostic factors of OS

As shown in **Table 2**, we included 11 variables of demographic and clinical characteristics in univariate Cox regression analysis. In the univariate analysis, all 11 variables were strongly associated with OS (*P* < 0.05). According to multivariate Cox regression analysis, histological type that was non-endometrioid, high tumor grade, absence of surgery, absence of radiotherapy, absence of chemotherapy, and presence of brain and liver metastases were all poor predictors of OS. However, age, race, marital status, bone metastasis and lymph node resection were no longer risk factors for EC patients with lung metastasis.

**Table 2:** Univariate and multivariate Cox analyses for OS in EC patients with lung metastasis.

	Univariate analysis		Multivariate analysis	
	HR(95% CI)	*P*	HR(95% CI)	*P*
Age (year)				
≤65	reference			
>65	1.255(1.144-1.377)	<0.001		
Race				
White	reference			
Black	1.332(1.184-1.498)	<0.001		
Others	0.975(0.831-1.143)	0.791		
Marital status				
Married	reference			
Single	1.239(0.999-1.274)	0.101		
Others	1.123(1.115-1.376)	0.001		
Histological type				
EEA	reference		reference	
SEA	1.068(0.935-1.221)	0.412	1.12(0.942-1.331)	0.196
Others	1.29(1.163-1.431)	<0.001	1.471(1.124-1.464)	<0.001
Grade				
I	reference		reference	
II	1.460(1.101-1.934)	0.027	1.358(0.897-2.054)	0.147
III/IV	2.295(1.799-2.928)	<0.001	2.062(1.443-2.945)	<0.001
Unknown	2.194(1.718-2.801)	<0.001	1.434(1.003-2.051)	0.001
Tumor size (cm)				
<5	reference		reference	
5-10	1.091(0.929-1.280)	0.37	1.050(0.830-1.408)	0.683
>10	1.511(1.258-1.814)	<0.001	1.243(0.950-1.628)	0.112
Unknown	1.556(1.348-1.795)	<0.001	0.868(0.697-1.082)	0.208
Surgery				
Yes	reference		reference	
No	2.433(2.210-2.678)	<0.001	2.546(2.161-3.001)	<0.001
Radiotherapy				
Yes	reference		reference	
No	1.161(1.044-1.292)	0.021	1.317(1.127-1.476)	<0.001
Chemotherapy				
Yes	reference		reference	
No	2.814(2.558-3.095)	<0.001	2.791(2.345-3.217)	<0.001
Brain metastasis				
No	reference		reference	
Yes	1.606(1.313-1.965)	<0.001	1.712(1.253-2.318)	<0.001
Liver metastasis				
No	reference		reference	
Yes	1.848(1.650-2.068)	<0.001	1.572(1.408-1.857)	<0.001
Bone metastasis				
No	reference			
Yes	1.411(1.253-1.588)	<0.001		
Lymph node metastasis				
Yes	reference			
No	1.464(1.272-1.684)	<0.001		

EEA, endometrial endometrioid adenocarcinoma; SEA, serous endometrioid adenocarcinoma.

### Nomogram construction and validation

**Figure 1:**
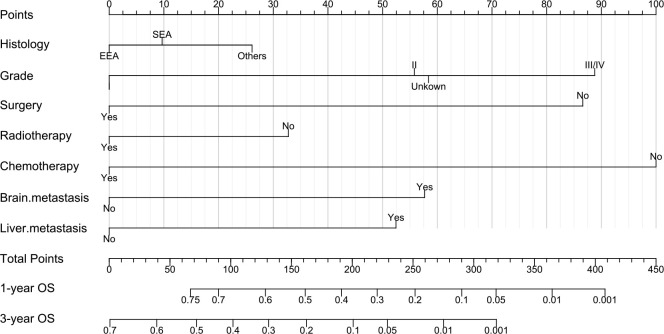
Nomogram to predict the 1-year and 3-year overall survival (OS) of EC patients with lung metastasis. EEA, endometrial endometrioid adenocarcinoma; SEA, serous endometrioid adenocarcinoma.

We constructed a nomogram model (**Figure 1**) using the variables of tumor grade, histological type, surgery, adjuvant chemotherapy, adjuvant radiation, and brain and liver metastases. In the nomogram, the corresponding number of points was assigned to a given magnitude of each variable, and the cumulative point score for all the variables was matched to a scale of the outcome (14), which predicted the 1-year and 3-year OS probabilities for EC patients with lung metastasis at the bottom. Measured by the standard deviation (SD) along the nomogram scales (15), chemotherapy, tumor grade, surgery and brain metastasis were the top four factors that impacted prognosis. The specific points of each variable in the nomogram are listed in **Table S2**.

**Figure 2:**
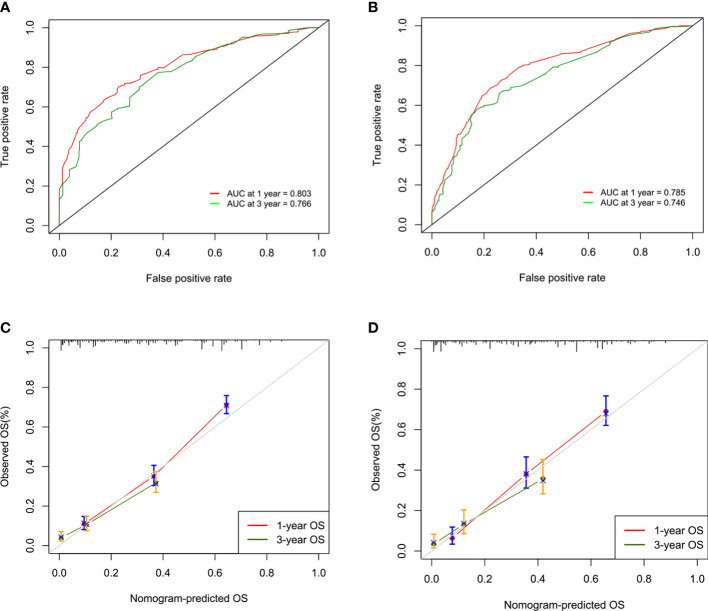
ROC curves and AUCs for 1-year and 3-year overall survival (OS) in the training cohort **(A)** and the validation cohort **(B)**. Calibration curves of 1-year and 3-year overall survival (OS) in the training cohort **(C)** and the validation cohort **(D)**.

The concordance indexes (C-indexes) were 0.750 (95% CI, 0.732-0.767) and 0.743 (95% CI, 0.719-0.767) for the training cohort and validation cohort, respectively, which showed the good discriminative ability of this nomogram. Moreover, the AUCs of the time-dependent ROC curves in the training cohort were 0.803 and 0.766 for 1-year and 3-year OS, respectively, and those in the validation cohort were 0.785 and 0.746 (**Figures 2A, B**), indicating that the nomogram model had stable discriminative ability. The calibration plot showed favorable consistency between the nomogram-estimated risk and the observed risk for 1-year and 3-year OS in both the training and validation cohorts (**Figures 2C, D**).

### Clinical utility of the nomogram

The DCA curves for the nomogram of the training and validation cohorts are shown in **Figure S1**. The nomogram had a positive clinical utility in predicting the OS at 1 year and 3 years, as it adds additional net benefits in both cohorts.

An online calculator of our nomogram is available on the internet at https://endometrialcancer.shinyapps.io/DynNomapp/. Predicted survival rates across time can be determined by entering the variables and follow-up time into the webserver, which provides convenient use and future validation for researchers and clinicians (**Figure S2**).

**Figure 3:**
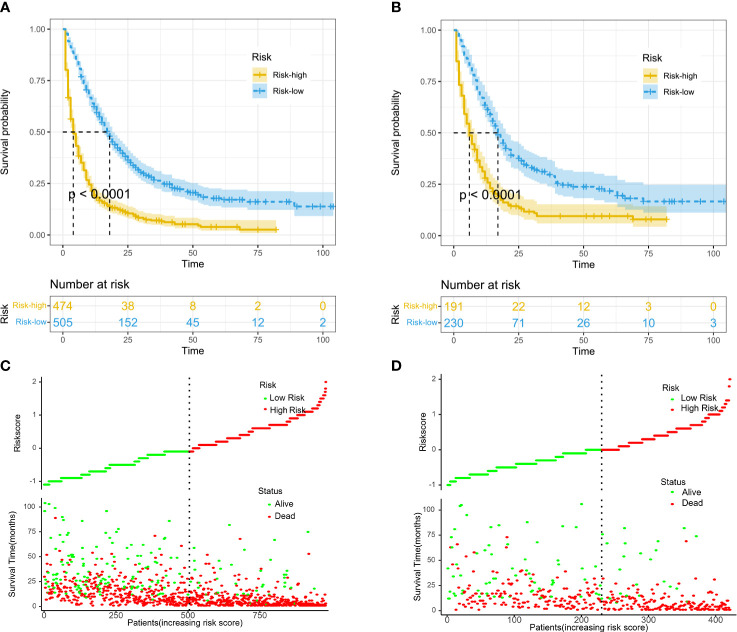
Kaplan–Meier curves of overall survival (OS) for risk stratification in the training cohort **(A)** and validation cohort **(B)**. Scatter diagram displaying the survival status, survival time, and risk score for risk stratification in the training cohort **(C)** and validation cohort **(D)**.

Finally, the patients were stratified into high-risk (risk score >150) and low-risk (risk score <150) groups according to the nomogram score. Kaplan–Meier survival curves were plotted and showed that patients in the high-risk group had significantly worse OS than those in the low-risk group (**Figures 3A, B**). In the scatter diagram based on the risk score, the number of deaths was increased and the survival time was clearly shorter in the high-risk group (**Figures 3C, D**).

## Discussion

Although two-thirds of women with EC present with stage I disease and most have a good prognosis, stage IVB women with advanced or metastatic disease are considered a high-risk population (16). A SEER database study of 2948 women with stage IV EC found that the most common metastatic site was the lung (37.0%) (8). Similarly, other studies confirmed that distant metastasis commonly occurs in the lung and lymph nodes (7, 17, 18). Lung metastasis was associated with better survival than other organ metastases. Less common sites of metastasis include the liver, bone and brain.

A previous report proposed that squamous epithelial differentiation, deep myometrial invasion, and large tumor size might be risk factors for lung metastasis in patients with EEC (19). Advanced stage, absence of adjuvant radiotherapy after initial surgery, absence of chemotherapy and lymphatic metastasis were identified as independent risk factors for poor prognosis after lung metastasis (20). In this population‐based analysis, clinicopathological factors, such as race, age, marital status, tumor grade, histological type, tumor size, lymph nodes resected and therapy, were integrated to predict OS in 1542 patients. Seven variables were selected by univariate and multivariate Cox regression based on the AIC minimum. Then, we constructed a nomogram to predict the prognosis of EC patients with lung metastasis, and the validation showed that our nomogram had good discriminative and calibration capabilities. The strongest influencing factor of prognosis was chemotherapy, followed by surgery, tumor grade and brain metastasis, and histological type had the least influence.

Surgery is the mainstay treatment for women with localized EC. However, surgery for metastatic or advanced EC is more controversial (18), and consideration should be given to the time from diagnosis, number of disease sites, location of recurrence, and patient performance status (16). The greatest benefit has been reported for those who undergo optimal surgical cytoreduction (21). The frequency of neoadjuvant chemotherapy and interval debulking surgery has increased significantly in the United States (22). Between 2010 and 2015, the number of patients receiving neoadjuvant chemotherapy prior to surgery for stage IVB EC increased from 11.6% to 21.7% (23). Tobias showed that women with metastatic EC who were treated with neoadjuvant chemotherapy may have superior survival for 3 to 8 months after the initiation of treatment. In contrast, women treated with debulking surgery are at increased risk of early death but have a more favorable long-term prognosis (2). In particular, surgery and adjuvant chemotherapy upon recurrence were associated with improved OS after lung metastasis (20, 24). In the present study, our nomogram showed that chemotherapy and surgery, which were the top two factors in the seven included variables, were both associated with improved OS for EC patients with lung metastasis, in line with the findings of a previous study. Analysis of the NCDB and SEER registries have demonstrated that use of multimodality treatment with radiation and chemotherapy was associated with improved OS compared to chemotherapy alone in women with stage IVB endometrial cancer. In addition, the use of chemotherapy with vaginal brachytherapy (VBT) was associated with improved OS compared to chemotherapy alone, and combination of chemotherapy with external beam radiation therapy (EBRT) +VBT was associated with the greatest OS benefit in women with stage IVB disease (25). Stereotactic body radiation therapy (SBRT) with daily online cone beam computed tomography for lung metastases achieved excellent local tumour control with encouraging 2 and 3 year survival (26). The appliance of chemotherapy after initial surgery was associated with a reduction in OS compared with radiotherapy in EC patients with lung metastasis (20). Our study identified adjuvant radiation is associated with improved survival, and further study to determine the role of combined chemotherapy and radiation is indicated.

Various studies have tried to identify the predictors of distant metastatic sites in stage IV EC (7, 8, 17). The findings of the current study showed that the liver was the most common metastatic organ (19.5%) in EC patients with lung metastasis. Liver and brain metastases were independent predictors associated with OS, whereas bone metastasis was not a significant independent risk factor. Mao found that in patients with two metastatic sites, the combination of lung and bone metastases had the longest median OS, and liver and brain metastases had the shortest median OS (6). If recurrence is only in the lung, survival is better compared with the patients who had extrapulmonary recurrence in addition to lung (20). In general, the patients with 3 or less metastatic lesions showed better survival than those with more than 3 metastases (27). By contrast, Turan et al (20) showed that number of tumoral nodules in the lung were not associated with OS.

Histopathologic grade was an independent prognostic factor for OS in the present study, and grade III/IV (poorly differentiated or undifferentiated) appeared to worsen long-term OS. This study also found that patients with non-endometrioid subtypes had shorter survival times than those with endometrioid subtypes, which is consistent with the findings of several retrospective studies (6, 8, 20). Age and race were not incorporated into the nomogram because of the increased AIC value. To the best of our knowledge, age was an independent prognostic factor in EC patients but was not a significant prognostic factor for OS in EC patients with lung metastasis in the current study and previous studies (20, 27). Wei Jiang reported that a large tumor size might be a risk factor for stage I EEC with lung metastasis (19), and another study showed that tumor size is not convenient or effective for predicting OS after pulmonary recurrence (20). In addition, 50.1% of the included population had missing information for tumor size in the present study. Due to the above reasons, tumor size may be a weak predictor of OS in EC patients with lung metastasis.

The C-indexes of the training and validation cohorts were 0.750 and 0.743, respectively. The training and validation cohorts, combined with the calibration plot, show that our nomogram has good discrimination and calibration capabilities. DCA curves are a new method for assessing whether nomogram-assisted decisions improve patient outcomes based on the threshold probability (14, 28). DCA curves showed that our nomogram had a positive benefit of clinical application. In the current data set, we divided the patients into a high-risk group and a low-risk group according to their nomogram score, and the Kaplan-Meier curves showed that the low-risk group exhibited significantly better OS than the high-risk group.

This study has several limitations. The main limitation is that T stage and M stage, overriding prognostic factors in EC, were not included in the nomogram since nearly half of the data were not available. Second, The Cancer Genome Atlas (TCGA) identified four molecular subtypes of EC (29), and the survival differences between the TCGA molecular subtypes have been replicated in later studies using clinically applicable methods (16, 18, 30). However, the SEER database does not provide the molecular classification of EC. Future prospective studies and clinical validation are still warranted to improve and confirm this nomogram.

In conclusion, our study suggests that tumor grade, histological type, surgery, adjuvant chemotherapy, adjuvant radiation, brain metastasis and liver metastasis are independent prognostic factors. A nomogram was developed and validated for predicting the survival for EC patients with lung metastasis. To facilitate clinical use of this nomogram, an online calculator is provided at https://endometrialcancer.shinyapps.io/DynNomapp/. Overall, our nomogram is an easy-to-use, highly accurate tool to predict the prognosis of EC patients with lung metastasis.

## Data availability statement

Publicly available datasets were analyzed in this study. This data can be found here: the SEER database.

## Author contributions

GY and YL designed the study and wrote the manuscript. YD collected the data from SEER database and contributed to the statistical analysis. XM performed figures and tables. YX and XZ contributed to the final data and manuscript revision. All authors contributed to the article and approved the submitted version.

## Funding

This study was supported by the Science and Technology Project of Hennan Province (LHGJ20210436) and Higher Education Key Scientific Research Plan of Henan Province(22A320007).

## Conflict of interest

The authors declare that the research was conducted in the absence of any commercial or financial relationships that could be construed as a potential conflict of interest.

## Publisher’s note

All claims expressed in this article are solely those of the authors and do not necessarily represent those of their affiliated organizations, or those of the publisher, the editors and the reviewers. Any product that may be evaluated in this article, or claim that may be made by its manufacturer, is not guaranteed or endorsed by the publisher.

## Supplementary material

Supplementary Figure 1 DCA curves of 1-year **(A)** and 3-year **(B)** overall survival (OS) in the training cohort. DCA curves of 1-year **(C)** and 3-year **(D)** overall survival (OS) in the validation cohort.

Supplementary Figure 2 Dynamic web version of the nomogram. The left side of the interface is the corresponding data, and the right side is the ‘Survival Plot’, ‘Predicted Survival’, Numerical Summary’ and ‘Model Summary’ modules.

Supplementary Table 1 Histological type of EC used in the analysis. ICD-O-3, International Classification of Disease for Oncology (after 2001).

Supplementary Table 2 Points of each variable in the nomogram. EEA endometrial endometrioid adenocarcinoma, SEA serous endometrioid adenocarcinoma, SD standard deviation.
