# Supplementary figures and images for: Survival nomogram for endometrial cancer with lung metastasis: A SEER database analysis
Source: Front Oncol. 2022 Oct 7;12:978140. doi: 10.3389/fonc.2022.978140 (PMC9585205; doi:10.3389/fonc.2022.978140)

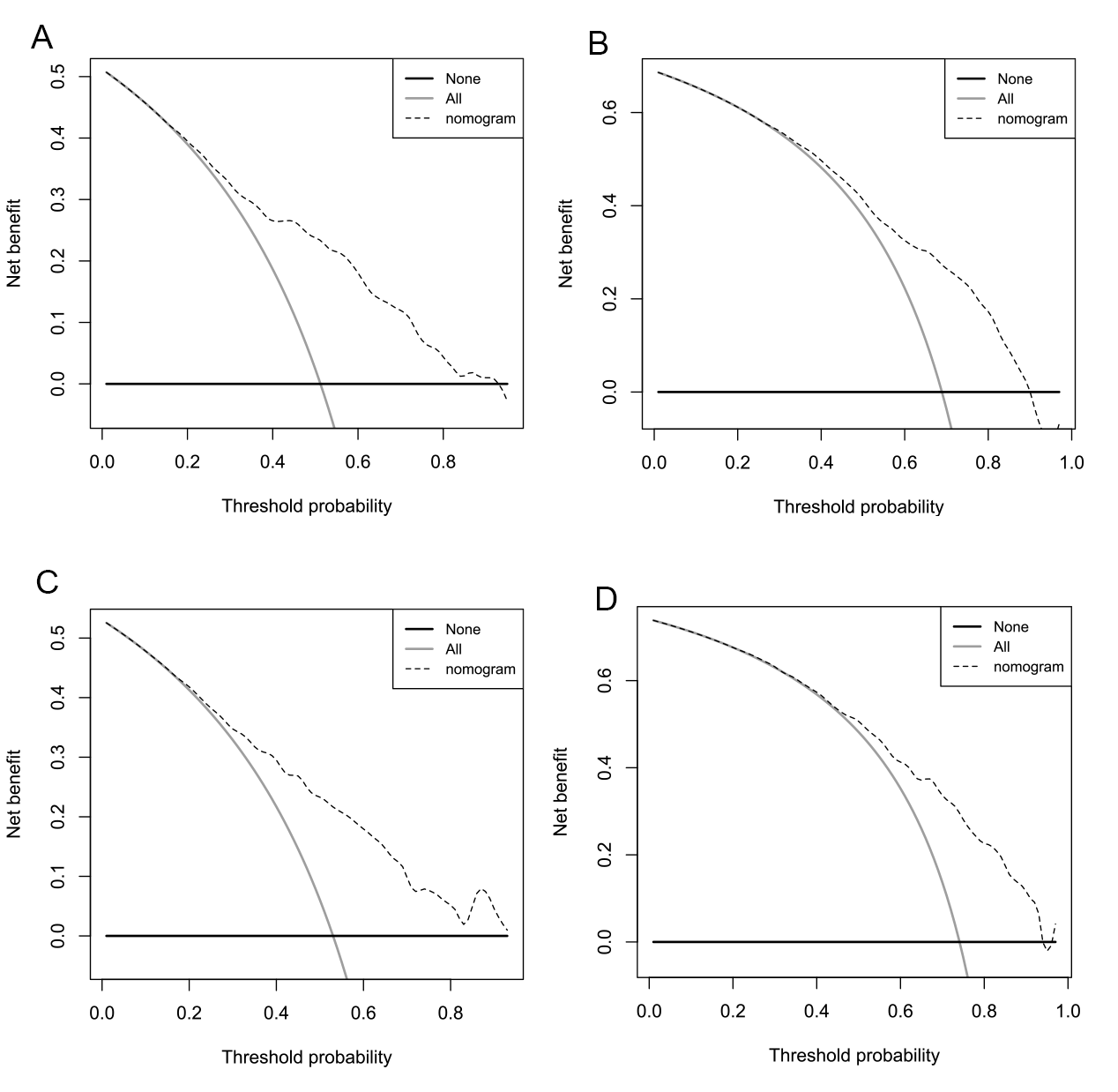

Supplement: Supplementary Figure 1 — DCA curves of 1-year (A) and 3-year (B) overall survival (OS) in the training cohort. DCA curves of 1-year (C) and 3-year (D) overall survival (OS) in the validation cohort. [file Image_1.tif]

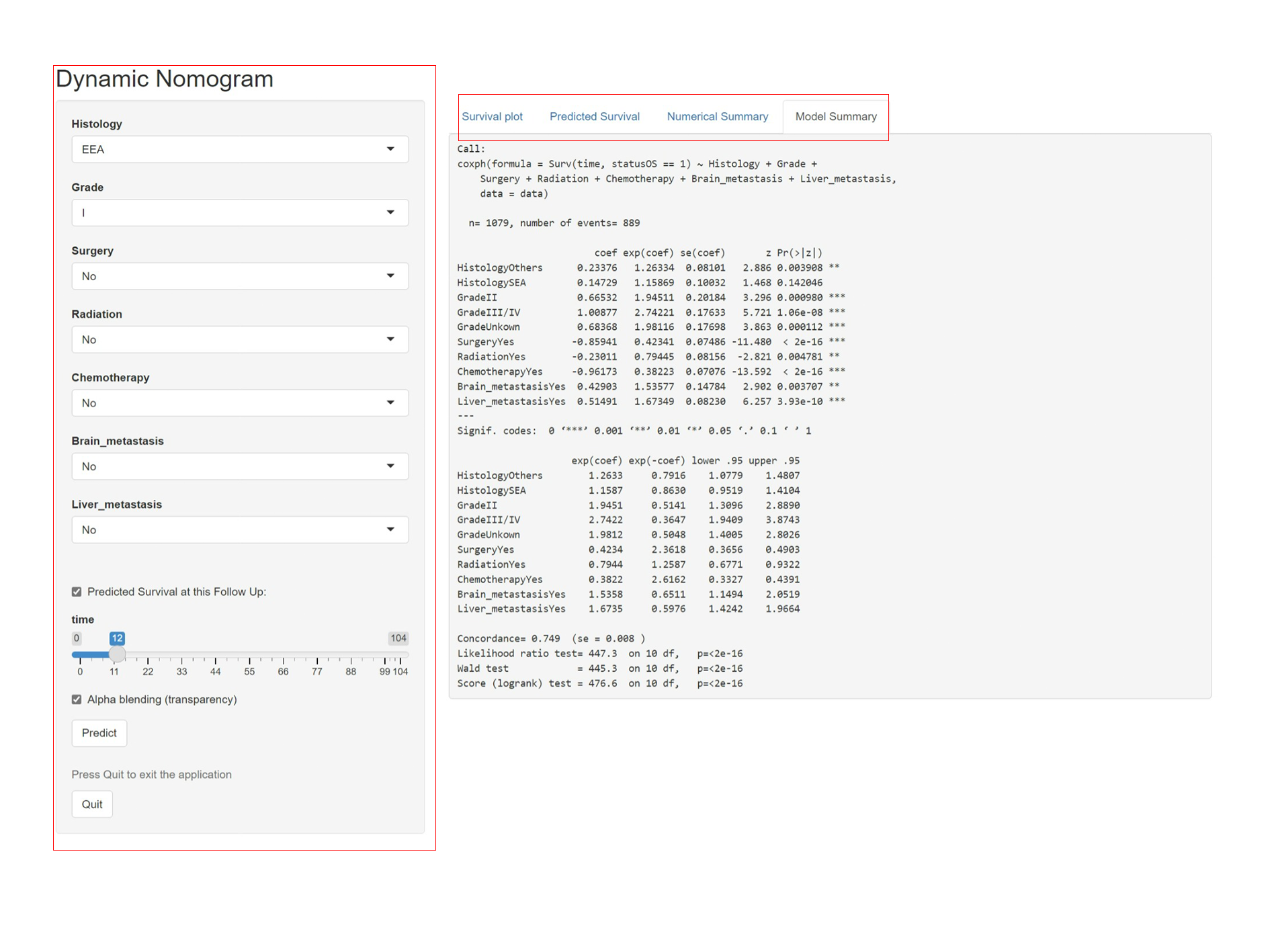

Supplement: Supplementary Figure 2 — Dynamic web version of the nomogram. The left side of the interface is the corresponding data, and the right side is the ‘Survival Plot’, ‘Predicted Survival’, Numerical Summary’ and ‘Model Summary’ modules. [file Image_2.tif]
